# Formant Space Reconstruction From Brain Activity in Frontal and Temporal Regions Coding for Heard Vowels

**DOI:** 10.3389/fnhum.2019.00032

**Published:** 2019-02-08

**Authors:** Alessandra Cecilia Rampinini, Giacomo Handjaras, Andrea Leo, Luca Cecchetti, Monica Betta, Giovanna Marotta, Emiliano Ricciardi, Pietro Pietrini

**Affiliations:** ^1^IMT School for Advanced Studies Lucca, Lucca, Italy; ^2^Department of Philology, Literature and Linguistics, University of Pisa, Pisa, Italy

**Keywords:** fMRI, language, speech, vowels, production, perception, tones, formants

## Abstract

Classical studies have isolated a distributed network of temporal and frontal areas engaged in the neural representation of speech perception and production. With modern literature arguing against unique roles for these cortical regions, different theories have favored either neural code-sharing or cortical space-sharing, thus trying to explain the intertwined spatial and functional organization of motor and acoustic components across the fronto-temporal cortical network. In this context, the focus of attention has recently shifted toward specific model fitting, aimed at motor and/or acoustic space reconstruction in brain activity within the language network. Here, we tested a model based on acoustic properties (formants), and one based on motor properties (articulation parameters), where model-free decoding of evoked fMRI activity during perception, imagery, and production of vowels had been successful. Results revealed that phonological information organizes around formant structure during the perception of vowels; interestingly, such a model was reconstructed in a broad temporal region, outside of the primary auditory cortex, but also in the *pars triangularis* of the left inferior frontal gyrus. Conversely, articulatory features were not associated with brain activity in these regions. Overall, our results call for a degree of interdependence based on acoustic information, between the frontal and temporal ends of the language network.

## Introduction

Classical models of language have long proposed a relatively clear subdivision of tasks between the inferior frontal and the superior temporal cortices, ascribing them to production and perception respectively (Damasio and Geschwind, 1984; Gernsbacher and Kaschak, 2003). Nevertheless, lesion studies, morphological and functional mapping of the cortex evoke a mixed picture concerning the control of perception and production of speech (Josephs et al., 2006; Hickok et al., 2011; Basilakos et al., 2015; Ardila et al., 2016; Schomers and Pulvermüller, 2016).

Particularly, classical theories propose that, on one hand, perception of speech is organized around the primary auditory cortex in Heschl’s gyrus, borrowing a large patch of superior and middle temporal regions (Price, 2012); on the other hand, production would be coordinated by an area of the inferior frontal cortex, ranging from the ventral bank of the precentral gyrus toward the *pars opercularis* and the *pars triangularis* of the inferior frontal gyrus, the inferior frontal sulcus, and, more medially, the insular cortex (Penfield and Roberts, 1959).

This subdivision, coming historically from neuropsychological evidence of speech disturbances (Poeppel and Hickok, 2004), makes sense when considering that the two hubs are organized around an auditory and a motor pivot (Heschl’s gyrus and the face-mouth area in the ventral precentral gyrus), although the issue of their exact involvement already surfaced at the dawn of modern neuroscience (Cole and Cole, 1971; Boller, 1978).

Eventually, the heightened precision of modern, *in vivo*, brain measures in physiology and pathology ended up supporting such a complex picture, since an exact correspondence of perception/production speech deficits with the classical fronto-temporal subdivision could not be validated by virtual lesion studies (Fadiga et al., 2002; D’Ausilio et al., 2009, 2012b). Moreover, cytoarchitecture, connectivity and receptor mapping results do suggest a fine-grained parcellation of frontal and temporal cortical regions responsible for speech (Catani and Jones, 2005; Anwander et al., 2006; Fullerton and Pandya, 2007; Hagmann et al., 2008; Amunts et al., 2010; Amunts and Zilles, 2012).

Functional neuroimaging and electrophysiology have therefore recently approached the issue of mapping the exact organization of the speech function, to characterize the fronto-temporal continuum in terms of cortical space-sharing [i.e., engagement of the same region(s) by different tasks] and neural code-sharing (i.e., similar information content across regions and tasks) (Lee et al., 2012; Tankus et al., 2012; Grabski et al., 2013; Arsenault and Buchsbaum, 2015; Correia et al., 2015; Cheung et al., 2016; Markiewicz and Bohland, 2016). Considering this, such studies seemingly align to phonological theory by validating perceptuo-motor models of speech (Schwartz et al., 2012; Laurent et al., 2017), where phonemes embed motor and acoustic information. In fact, vowels are indeed represented by a model based on harmonic properties (formants) modulated by tongue-lip positions: such a model is by all means based on acoustics, but it is also tightly linked to articulation (Ladefoged and Disner, 2012).

Previous fMRI attempts have been made to reconstruct formant space in the auditory cortex (Formisano et al., 2008; Bonte et al., 2014) with a model restricted to a subsample of vowels lying most distant in a space defined by their harmonic structure. Electrocorticographic recordings have also shown similar results and demonstrated the fine-tuning of the temporal cortex to harmonic structure (Chang et al., 2010; Mesgarani et al., 2014; Chakrabarti et al., 2015). In fact, the possibility of mutual intelligibility along the production-perception continuum, if demonstrated through shared encoding of neural information, might enrich the debate around the neurofunctional correlates of the motor theory of speech perception (MTSP; Liberman et al., 1967), and, more generally, action-perception theories (Galantucci et al., 2006).

In a previous study, a searchlight classifier on fMRI data obtained during listening, imagery and production of the seven Italian vowels, revealed that both the temporal and frontal hubs are sensitive to perception and production, each engaging in their classical, as well as non-classical function (Rampinini et al., 2017). Particularly, though, vowel-specific information was decoded in a spatially and functionally segregated fashion: in the inferior frontal cortex, adjoining regions engaged in vowel production, motor imagery and listening along a postero-anterior axis; in the superior temporal cortex, the same pattern was observed when information relative to perception and motor imagery of vowels was mapped by adjoining regions. Moreover, results from a control task of pure tone perception highlighted the fact that tone sensitivity was also present in the superior temporal and inferior frontal cortices, suggesting a role for these regions in processing low-level, non-strictly linguistic information.

Despite evidence of functional and spatial segregation across the fronto-temporal speech cortex down to the phonological level, a question remained unsolved: which features in the stimuli better describe brain activity in these regions? To investigate this issue, we sought to reconstruct formant and motor spaces from brain activity within each set of regions known to perform listening, imagery and production of the seven Italian vowels, using data acquired in our previous fMRI study and a multivariate procedure based on canonical correlation (Bilenko and Gallant, 2016).

## Materials and Methods

### Formant Model

The seven vowels of the Italian language were selected as experimental stimuli (IPA: [i] [e] [ε] [a] [ɔ] [o] [u]). While pure tones do not retain any harmonic structure, vowels are endowed with acoustic resonances, due to the modulation of the glottal signal by the vocal tract acting as a resonance chamber. Modulation within the phonatory chamber endows the glottal signal (F0), produced by vocal fold vibration, with formants, i.e., harmonics rising in average frequency as multiples of the glottal signal. Along the vertical axis, first-formant (F1) height correlates inversely with tongue height: therefore, the lower one’s tongue, the more open the vowel, the higher frequency of the first formant. The second formant (F2) instead correlates directly with tongue advancement toward the lips. Formant space for the Italian vowels makes it so that each vowel is described by the joint and unique contribution of its first and second formant (Albano Leoni and Maturi, 1995): when first and second formant are represented one as a function of the other, their arrangement in formant space resembles a trapezoidal shape.

Three recordings of each vowel (21 stimuli, each lasting 2 s) were obtained using Praat (©Paul Boersma and David Weenink,^1^) from a female, Italian mother-tongue speaker (44100 Hz frequency sampling rate; F0: 191 ± 2.3 Hz). In Praat, we generated spectrograms for each vowel so as to obtain formant listings for F1 and F2, with a time step of 0.01 ms and a frequency step of 0.05 Hz. Average F1 and F2 were obtained by mediating all sampled values within-vowel and are reported, together with the corresponding standard deviations, in Table 1 and Figure 3. These values were converted from Hertz to Bark and subsequently normalized: eventually, they defined the formant model.

**Table 1:** Average F1 and F2 values and standard deviations for each stimulus.

*Vowel*	*F1(Hz)*	*F2(Hz)*
*i*	305 ± 21.1	2170 ± 25.7
*e*	303 ± 35.9	1736 ± 30.7
*e*	400 ± 27.1	1428 ± 47.4
*a*	525 ± 28.9	1139 ± 7.1
*ɔ*	455 ± 68.1	836 ± 34.9
*o*	338 ± 23.4	637 ± 71.6
*u*	278 ± 16.2	604 ± 27.0

### Articulatory Model

Structural images of the original speaker’s head were used to construct a model based on measurements of the phonatory chamber as in Laukkanen et al. (2012), while the speaker pronounced the vowels. Structural imaging of the speaker uttering three repetitions of each vowel was obtained in a separate session from auditory recording. The speaker was instructed to position her mouth for the selected vowel right before the start of each scan, so as to image steady-state articulation. Scanning parameters were aimed at capturing relevant structures in the phonatory chamber; at the same time, each sequence needed to last as long as the speaker could maintain constant, controlled airflow while keeping motion to a minimum: with this goal, scanning time for each vowel lasted 21 s. Structural T1-weighted images were acquired on a Siemens Symphony 1.5 Tesla scanner, equipped with a 12-channel head coil (TR/TE = 195/4.76 ms; FA = 70°; matrix geometry: 5 × 384 × 384, sagittal slices, partial coverage, voxel size 5 mm × 0.6 mm × 0.6 mm, plus 1 mm gap).

**Figure 1:**
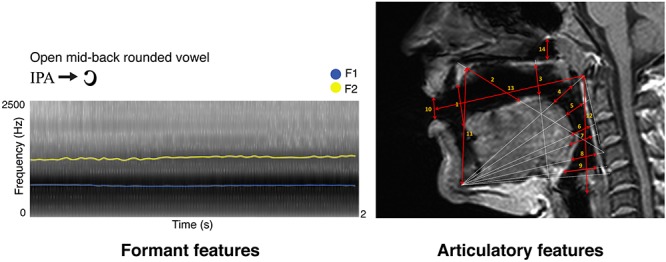
Here we show a sample vowel by its formant **(left)** and articulatory **(right)** representations, as described in Materials and Methods. Formant features represent F1 in blue and F2 in yellow (sampled time step = 0.025 s for display purposes; frequency step unaltered). On the top right, MRI-based articulatory features for the same vowel are indicated by red arrows, with numbers matching the anatomical description of the same measure in Materials and Methods.

Three independent raters performed the MRI anatomical measurements. Particularly, fourteen distances were measured in ITK-SNAP (Yushkevich et al., 2006) as follows: (1) we measured from the tip of the tongue to the anterior edge of the alveolar ridge; (2) we connected the anterior edge of the hard palate to the anterior upper edge of the fourth vertebra, and in that direction we measured from the anterior part of the hard palate to the dorsum of the tongue; (3) we connected the lowermost edge of the jawbone contour to the upper edge of the fifth vertebra, and in that direction we measured from the posterior dorsum of the tongue, to the posterior edge of the hard palate, at a 90° angle with the direction line; (4) we connected the lowermost edge of the jawbone contour to the anterior edge of the Arch of Atlas, and in that direction we measured from the anterior tongue body to the soft palate; (5) we connected the lowermost edge of the jawbone contour to half the distance between the anterior edge of the arch of Atlas and the upper edge of the third vertebra, and in that direction we measured from the posterior tongue body to the back wall of the pharynx; (6) we connected the lowermost edge of the jawbone contour to the upper edge of the third vertebra, and in that direction we measured from the upper tongue root to the back wall of the pharynx; (7) we connected the lowermost edge of the jawbone contour to the longitudinal midpoint of the third vertebra, and in that direction we measured from the lowermost tongue root to the lowermost back wall of the pharynx; (8) we connected the lowermost edge of the jawbone contour to the anterior upper edge of the fourth vertebra and in that direction we measured from the epiglottis to the back wall of the pharynx; (9) we connected the lowermost edge of the jawbone contour and the anterior lower edge of the fourth vertebra, and in that direction we measured from the root of the epiglottis to the back wall of the pharynx; (10) we measured lip opening by connecting the lips at their narrowest closure point; (11) we measured jaw opening by connecting the lowermost edge of the jawbone contour to the anterior end of the hard palate; (12) we measured the vertical extension of the entire vocal tract by tracing the distance between the posterior end of the vocal folds to the anterior lower arch of Atlas; (13) we measured the horizontal extension of the entire vocal tract by tracing the distance between the anterior arch of Atlas to the narrowest closure point between the lips; (14) in the naso-pharynx, we traced the distance between the highest point of the velum platinum and the edge of the sphenoid bone. As an example, Figure 1 reports the spectrogram of a vowel obtained in Praat and the MRI measurements of the phonatory chamber for the same vowel, according to Laukkanen et al. (2012).

Each rater produced a matrix of 21 rows (i.e., seven vowels with three repetitions each) and 14 columns (i.e., the fourteen anatomical distances). For each rating matrix, a representational dissimilarity matrix (RDM, cosine distance) was obtained, and subsequently the accordance (i.e., Pearson’s correlation coefficient) between the three RDMs was calculated to assess inter-rater variability. Furthermore, the three RDMs were averaged and non-metric multidimensional scaling was performed to reduce the original 14-dimensional space into two dimensions, thus approximating the dimensionality of the formant model. Finally, the two-dimensional matrix was normalized and aligned to the formant model (procrustes analysis using the rotational component only), to define the articulatory model as reported in Figure 3.

### Subjects

Fifteen right-handed (Edinburgh Handedness Inventory; laterality index 0.79 ± 0.17) healthy, mother-tongue Italian monolingual speakers (9F; mean age 28.5 ± 4.6 years) participated in the fMRI study, approved by the Ethics Committee of the University of Pisa.

### Stimuli

The seven vowels of the Italian language recorded during the experimental session, for the calculation of the formant model, were used as experimental stimuli (IPA: [i] [e] [ε] [a] [ɔ] [o] [u]). Moreover, by dividing the minimum/maximum average F1 range of the vowel set into seven bins, we also selected seven pure tones (450, 840, 1370, 1850, 2150, 2500, 2900 Hz), whose frequencies in Hertz were converted first to the closest Bark scale value, and then back to Hertz: this way, pure tones were made to fall into psychophysical sensitive bands for auditory perception. Then, pure tones were generated in Audacity (©Audacity Team,^2^; see Rampinini et al., 2017 for further details).

### Experimental Procedures

Using Presentation, we implemented a slow event-related paradigm (©Neurobehavioral Systems, Inc.,^3^) comprising two perceptual tasks defined as tone perception and vowel listening, a vowel articulation imagery task and a vowel production task. In perceptual trials, stimulus presentation lasted for 2 s and was followed by 8 s rest. Imagery/production trials started with 2 s stimulus presentation, then followed by 8 s maintenance phase, 2 s task execution (articulation imagery, or production of the same heard vowel) and finally 8 s rest. Globally, functional scans lasted 47 m, divided into 10 runs. All vowels and tones were presented twice to each subject, and their presentation order was randomized within and across tasks and subjects.

Functional imaging was carried out through GRE-EPI sequences on a GE Signa 3 Tesla scanner equipped with an 8-channel head coil (TR/TE = 2500/30 ms; FA = 75°; 2 mm isovoxel; geometry: 128 × 128 × 37 axial slices). Structural imaging was provided by T1-weighted FSPGR sequences (TR/TE = 8.16/3.18 ms; FA = 12°; 1mm isovoxel; geometry: 256x256x170 axial slices). MR-compatible on-ear headphones (30 dB noise-attenuation, 40 Hz to 40 kHz frequency response) were used to achieve auditory stimulation.

### fMRI Pre-processing

Functional MRI data were preprocessed using the AFNI software package, by performing temporal alignment of all acquired slices within each volume, head motion correction, spatial smoothing (4 mm FWHM Gaussian filter) and normalization. We then identified stimulus-related BOLD patterns by means of multiple linear regression, including movement parameters and signal trends as regressors of no interest (Rampinini et al., 2017). In FSL (Smith et al., 2004; Jenkinson et al., 2012) *T*-value maps of BOLD activity related to auditory stimulation (vowels, tones) or task execution (imagery, production) were warped to the Montreal Neurological Institute (MNI) standard space, according to a deformation field provided by the non-linear registration of T1 images of the same standards.

### Previously Reported Decoding Analysis

In our previous study, this dataset was analyzed to uncover brain regions involved in the discrimination of the four sets of stimuli. Using a multivariate decoding approach based on four searchlight classifiers (Kriegeskorte et al., 2006; Rampinini et al., 2017), we identified, within a pre-defined mask of language-sensitive cortex from the Neurosynth database (Yarkoni et al., 2011), a set of regions discriminating among seven classes of stimuli: the seven tones in the tone perception task and the seven vowels in the listening, imagery and production tasks (*p* < 0.05, corrected for multiple comparisons; see Figure 1). Moreover, accuracies emerging from the tone perception classifier had been used to measure sensitivity to low-level features of acoustic stimuli within regions identified by the vowel classifiers.

### Reconstructing Formant and Motor Features From Brain Activity

While a multivariate decoding approach had successfully detected brain regions representing vowels, it lacked the ability to recognize the specific, underlying information encoded in those regions, as previous evidence from fMRI had hinted (Formisano et al., 2008; Bonte et al., 2014). We therefore tested here whether the formant and articulatory models were linearly associated to brain responses in the sets of regions representing listened, imagined and produced vowels, as well as pure tones. To this aim, instead of adopting a single-voxel encoding procedure (Naselaris et al., 2011), we selected Canonical Correlation Analysis (CCA; Hotelling, 1936; Bilenko and Gallant, 2016) as a multi-voxel technique which provided a set of canonical variables maximizing the correlation between the two input matrices, X (frequencies of the first two formants of our recorded vowels or, alternatively, the two dimensions extracted from the vocal tract articulatory parameters) and Y (brain activity in all the voxels of a region of interest). Specifically, in the formant model, the X matrix described our frequential, formant-based model in terms of F1 and F2 values of the vowel recordings (three for each vowel, as described in the Stimuli paragraph), whereas, in the articulatory model, the X matrix described the phonatory chamber measurements extracted from structural MRI acquired during vowel articulation. The Y matrix instead consisted of the elicited patterns of BOLD activity, normalized within each voxel of each region. Since Y was a non full-rank matrix, Singular-Value Decomposition (SVD) was employed before CCA. In details, for each brain region and subject, the rank of Y was reduced by retaining the first eigenvectors to explain at least 90% of total variance. Subsequently, for each region and within each subject, a leave-one-stimulus-out CCA was performed (Bilenko and Gallant, 2016) thus to obtain two predicted canonical components derived from BOLD activity maximally associated to the two two-dimensional models. Afterward, predicted dimensions were aligned to the models (procrustes analysis using the rotational component only), and aggregated across subjects in each brain region. As a goodness-of-fit measure, R^2^ was computed between group-level predicted dimensions and the models. For the formant model, the predicted formants were converted back to Hertz and mapped in the F1/F2 space (Figure 3).

The entire CCA procedure was validated by a permutation test (10,000 permutations): specifically, at each iteration, the labels of brain activity patterns (i.e., the rows of the Y matrix, prior to SVD) were randomly shuffled and subjected to a leave-one-stimulus-out CCA in each subject. This procedure provided a null R^2^ distribution related to the group-level predicted dimensions. A one-sided rank-order test was carried out to derive the *p*-value associated with the original R^2^ measure (Tables 2–5). Subsequently, *p*-values were corrected for multiple comparisons by dividing the raw *p*-values by number of tests (i.e., six regions and three tasks, 18 tests).

**Table 2:** CCA results in regions from vowel listening, imagery and perception (lines), between brain activity in each task (columns) and the formant model.

	Region	Brain Activity		
		Vowel Listening	Vowel Imagery	Vowel Production
**Vowel Listening**	***Left pSTS-MTG***	***R^2^*** = **0.402**, ***p*** = **0.0001**	*R^2^* = 0.210, *p* = 0.0876	*R^2^* = 0.011, *p* = 0.7599
	***Left IFGpTri***	***R^2^*** = **0.391**, ***p*** = **0.0001**	*R^2^* = 0.165, *p* = 0.1826	*R^2^* = 0.125, *p* = 0.3244
*Vowel Imagery*	*Left pMTG-STG*	*R^2^* = 0.159, *p* = 0.2418	*R^2^* = 0.291, *p* = 0.0222	*R^2^* = 0.113, *p* = 0.4285
	*Right IFS-MFG*	*R^2^* = 0.234, *p* = 0.0706	*R^2^* = 0.248, *p* = 0.0572	*R^2^* = 0.334, *p* = 0.0074
	*Left IFS-MFG*	*R^2^* = 0.133, *p* = 0.2845	*R^2^* = 0.096, *p* = 0.3985	*R^2^* = 0.310, *p* = 0.0124
*Vowel Production*	*Left IFS-IFGpOp*	*R^2^* = 0.090, *p* = 0.4492	*R^2^* = 0.090, *p* = 0.4551	*R^2^* = 0.262, *p* = 0.0359

*R^2^ values and raw p-values were reported in the table. Please note that the statistical significance threshold after correction for multiple comparisons (i.e., Bonferroni) is 0.05/18 = 0.0028. Significant values are in bold font.*

**Table 3:** CCA results in tone perception regions, between vowel listening brain data and the formant model at group level.

Region		Brain Activity
		VOWEL LISTENING
*Tone Perception*	*Left STS*	*R^2^* = 0.169, *p* = 0.3077
	*Left IFG*	*R^2^* = 0.079, *p* = 0.6086
	*Right IFG*	*R^2^* = 0.185, *p* = 0.1852

*No *R^*2*^* value reached significance here.*

**Table 4:** CCA results in regions from vowel listening, imagery and perception (lines), between brain activity in each task (columns) and the articulatory model.

Region		Brain Activity		
		Vowel Listening	Vowel Imagery	Vowel Production
*Vowel Listening*	*Left pSTS-MTG*	*R^2^* = 0.317, *p* = 0.0067	*R^2^* = 0.250, *p* = 0.0399	*R^2^* = 0.106, *p* = 0.4195
	*Left IFGpTri*	*R^2^* = 0.283, *p* = 0.0179	*R^2^* = 0.068, *p* = 0.5224	*R^2^* = 0.090, *p* = 0.4515
*Vowel Imagery*	*Left pMTG-STG*	*R^2^* = 0.091, *p* = 0.4905	*R^2^* = 0.256, *p* = 0.0649	*R^2^* = 0.128, *p* = 0.3626
	*Right IFS-MFG*	*R^2^* = 0.182, *p* = 0.1658	*R^2^* = 0.320, *p* = 0.0099	*R^2^* = 0.299, *p* = 0.0189
	*Left IFS-MFG*	*R^2^* = 0.130, *p* = 0.2617	*R^2^* = 0.107, *p* = 0.3546	*R^2^* = 0.292, *p* = 0.0159
*Vowel Production*	*Left IFS-IFGpOp*	*R^2^* = 0.120, *p* = 0.3426	*R^2^* = 0.072, *p* = 0.4825	*R^2^* = 0.269, *p* = 0.0209

*R^*2*^ values and raw *p*-values were reported in the table. Please note that the statistical significance threshold after correction for multiple comparisons (i.e., Bonferroni) is 0.05/18 = 0.0028. No *R^*2*^* value survived correction for multiple comparisons here.*

**Table 5:** CCA results in tone perception regions, between vowel listening brain data and the articulatory model at group level.

Region		Brain Activity
		VOWEL LISTENING
*Tone Perception*	*Left STS*	*R^2^* = 0.120, *p* = 0.4952
	*Left IFG*	*R^2^* = 0.085, *p* = 0.5834
	*Right IFG*	*R^2^* = 0.184, *p* = 0.1858

*No *R^*2*^* value reached significance here.*

Finally, in regions surviving Bonferroni correction, confidence intervals (CI, 5^th^–95^th^ percentiles) were calculated trough a bootstrapping procedure by sampling the predicted dimensions across subjects (1000 iterations). In regions surviving Bonferroni correction, the comparison between the formant and articulatory models was achieved by comparing the two bootstrap distributions while maintaining the bootstrap scheme fixed, then measuring the 5^th^ and 95^th^ CI of the distribution obtained by computing their difference; such difference should not cross the zero-threshold to be significant (i.e., less than a 5% chance that the CI includes 0).

### Vowel Synthesis From Brain Activity

Using the predicted formants, we reconstructed the Italian vowels from brain activity. Specifically, we fed a two-column matrix containing predicted F1 and F2 values to the Vowel Editor program in the Praat suite (Boersma, 2006), which was able to synthesize waveforms of the seven vowels. Moreover, we also re-synthesized the spoken vowels (i.e., the original stimuli) to offer a direct comparison between natural and reconstructed speech (see Supplementary Material).

## Results

### Previous Results

**Figure 2:**
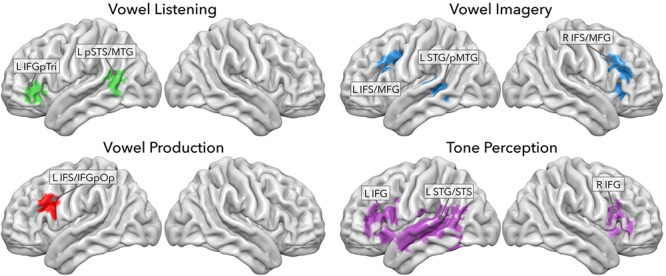
Searchlight classifier results from Rampinini et al. (2017). Each panel shows regions where model-free decoding was successful in each task.

In a previous study, we sought to decode model-free information content from regions involved in vowel listening, imagery and production, and in tone perception (Rampinini et al., 2017). Using four searchlight classifiers of fMRI data, we extracted a set of regions performing above-chance classification of seven vowels or tones in each task. As depicted in Figure 2, vowel listening engaged the *pars triangularis* of the left inferior frontal gyrus (IFGpTri), extending into the *pars orbitalis*. Vowel imagery engaged the bilateral inferior frontal sulcus (IFS) and intersected the middle frontal gyrus (MFG), slightly overlapping with the insular cortex (INS) as well. Production engaged the left IFS though more posteriorly into the sulcus, extending into the *pars opercularis* of the IFG (IFGpOp), and the MFG. In the temporal cortex, vowel listening engaged the left posterior portion of the superior temporal sulcus and middle temporal gyrus (pSTS-pMTG). Vowel imagery as well engaged a bordering portion of the left pMTG extending superiorly into the superior temporal gyrus (STG) and superior temporal sulcus (STS), while no temporal regions were able to disambiguate vowels significantly during overt production. A small cluster of voxels in the IFS/MFG was shared by vowel imagery and production, as well as another very small one in the middle temporal gyrus (MTG) was shared by imagery and listening. Further testing revealed that the imagery-sensitive left pMTG-STG region also represented pure tones, as well as IFGpTri during vowel listening, while the shared clusters in the IFS-MFG and MTG did not share tone representations.

### Model Quality Assessment

**Figure 3:**
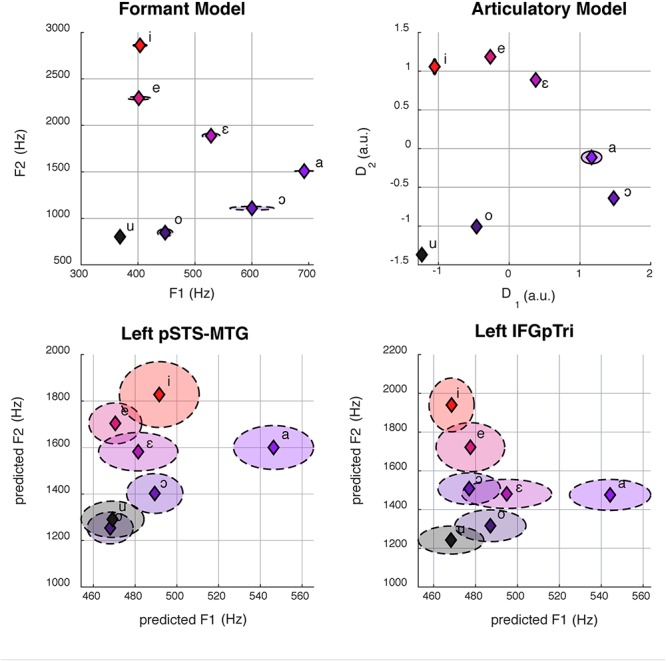
Here we show formant space **(top left)** and articulatory space **(top right)**. The bottom panel shows the reconstruction of formant space **(bottom left and right)** from group-level brain activity in the left pSTS-MTG (center, *R*^2^ = 0.40) and IFGpTri (right, *R*^2^ = 0.39) through CCA. Dashed ellipses represent standard errors. Articulatory space reconstruction is not reported for lack of statistical significance.

The articulatory model was constructed by three independent raters, who exhibited an elevated inter-rater accordance (mean = 0.94, min = 0.91, max = 0.96). As depicted in Figure 3, both models retain low standard errors between repetitions of the same vowel. Despite the high collinearity between the two models (*R*^2^ = 0.90), some discrepancies in the relative distance between vowels can be appreciated in Figure 3.

### Current Results

Here, we employed CCA to assess whether formant and articulatory models, derived from the specific acoustic and articulation properties of our stimuli, could explain brain activity in frontal and temporal regions during vowel listening, articulation imagery, and production. We correlated the formant and articulatory models to brain activity in a region-to-task fashion, i.e., vowel listening activity in vowel listening regions, imagery activity in imagery regions, and production activity in production regions; moreover, we correlated the models to brain activity from each task, in regions pertaining to all the other tasks (e.g., we tested vowel listening brain data for correlation with the formant and articulatory models not only in vowel listening regions, but also in imagery and production regions). Moreover, brain activity evoked by vowel listening was correlated with the two models in tone perception regions.

### Formant Model

Globally, the correlation between formant model and brain activity was significant at group level for vowel listening data, in vowel listening regions (uncorrected *p* = 0.0001; *Bonferroni-corrected p* < 0.05). As reported in Table 2, the left pSTS-MTG yielded an R^2^ of 0.40 (CI 5^th^–95^th^: 0.24–0.52) and left IFGpTri yielded an R^2^ of 0.39 (CI 5^th^–95^th^: 0.20–0.53). For these two regions a reconstruction of vowel waveforms from brain activity was also accomplished (see Supplementary Material). The correlation between formant model and brain data did not reach significance in any other tasks and regions after correction for multiple comparisons. In tone perception regions (i.e., left STG/STS, left IFG and right IFG, see Figure 2), the correlation between formant model and brain data did not reach significance (Table 3).

### Articulatory Model

**Figure 4:**
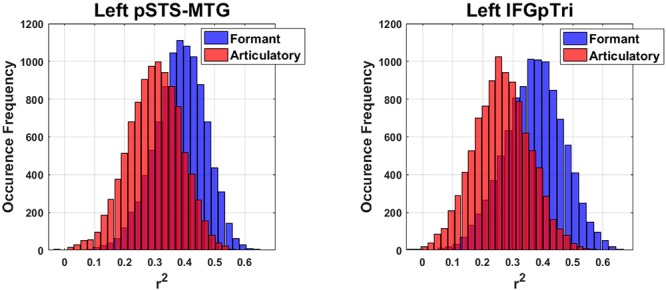
Bootstrap-based performance comparison between the articulatory and formant models, in regions surviving Bonferroni correction (C.I.: 5–95th of the distribution obtained by computing their difference).

Globally, the correlation between articulatory model and brain data did not survive correction for multiple comparisons in any tasks or regions. More importantly, comparison of the formant and motor bootstrap distributions revealed that the acoustic model fit significantly better than the motor model with brain activity in both left pSTS-MTG and left IFGpTri (*p* < 0.05; pSTS-MTG CI 5^th^–95^th^: 0.01–0.17; IFGpTri CI 5^th^–95^th^: 0.04–0.18; Figure 4). Articulatory model correlation with vowel listening brain activity in tone perception regions did not reach statistical significance (Table 5).

## Discussion

### General Discussion

Model-free decoding of phonological information from our previous study, provided a finer characterization of how production and perception of low-level speech units (i.e., vowels) do organize across a wide patch of cortex (Rampinini et al., 2017). Here, we extended those results by testing a frequential, formant-based model and a motor, articulation-based model on brain activity elicited during listening, imagery and production of vowels. As a result, we demonstrated that harmonic features (formant model) correlate with brain activity elicited by vowel listening, in the superior temporal sulcus and gyrus as shown in previous fMRI evidence (Formisano et al., 2008; Bonte et al., 2014). Importantly, here we show that a sub-region of the inferior frontal cortex, the *pars triangularis*, is tuned to formants, during vowel listening only. None of the other tasks reflected the formant model significantly, other than IFGpTri-listening and pSTS-MTG-listening. Moreover, despite the high collinearity between the two models, the performance of the articulatory model was never superior to that of the formant model.

### Model Fitting and the Perception-Production Continuum

The organization of speech perception and production in the left hemisphere has long been debated in the neurosciences of language. In fact, the fronto-temporal macro-region seems to coordinate in such a way that, on one hand, the inferior frontal area performs production-related tasks, as expected from its “classical” function (Dronkers, 1996; Skipper et al., 2005; Davis et al., 2008; Papoutsi et al., 2009), while also being engaged in perception tasks (Reiterer et al., 2008; Iacoboni, 2008; Flinker et al., 2015; Cheung et al., 2016; Rampinini et al., 2017); in turn, the superior temporal area, classically associated to perception (Evans and Davis, 2015; Zhang et al., 2016; Feng et al., 2017), seems to engage in production as well, despite the topic having received less attention in literature (Okada and Hickok, 2006; Arsenault and Buchsbaum, 2015; Evans and Davis, 2015; Rampinini et al., 2017; Skipper et al., 2017). Finally, sensitivity to tones seems to engage sparse regions across the fronto-temporal speech cortex (Reiterer et al., 2008; Rampinini et al., 2017). This arrangement of phonological information, despite being widely distributed along the fronto-temporal continuum, seems characterized by spatial and functional segregation (Rampinini et al., 2017). Our previous results suggested interesting scenarios as to what “functional specificity” means: in this light, we hypothesized that a model fitting approach would provide insights on the representation of motor or acoustic information in those regions. Therefore, in this study, we assessed whether formant and/or articulatory information content is reflected in brain activity, in regions involved in listening and production tasks, already proven to retain a capacity for vowel discrimination.

It is common knowledge in phonology that a perceptuo-motor model, i.e., a space where motor and acoustic properties determine each other within the phonatory chamber, describes the makeup of vowels (Stevens and House, 1955; Ladefoged and Disner, 2012; Schwartz et al., 2012). This premise could have led to one of the following: in a scenario, formant and articulatory information could have been detected in brain activity on an all-out shared basis; therefore, data from all tasks could have reflected both models in their own regions and those from all other tasks, confirming that the acoustic and motor ends of the continuum indeed weigh the same in terms of cortical processing. In another scenario, a specific task-to-region configuration could have been detected, where information in listening and production regions reflected the formant and articulatory model, respectively. An all-out sharing of formant and articulatory information (former scenario) would have pointed at an *identical* perceptuo-motor model being represented in regions involved in *different* tasks. A specific task-to-region scenario, instead, would have pointed at a subdivision of information that completely separates listened vowels from imagined or produced ones. Yet again, experimental phonology has long argued in favor of an elevated interdependence between the formant and articulatory models (Stevens and House, 1955; Moore, 1992; Dang and Honda, 2002), which is not new to neuroscience either, with data showing perception-related information in the ventral sensorimotor cortex and production-related information in the superior temporal area (Arsenault and Buchsbaum, 2015; Cheung et al., 2016). Thus, it seemed reasonable to hypothesize a certain degree of mutual intelligibility between the frontal and temporal hubs, even maintaining that the two ends of the continuum retain their own specificity of function (Hickok et al., 2011; D’Ausilio et al., 2012a). To what extent though, it remained to be assessed.

In our results, vowel listening data reflected the formant model in a temporal *and* in a frontal region, providing a finer characterization of how tasks are co-managed by the temporal and frontal ends of the perception-production continuum, in line with the cited literature. Particularly, formant space was reconstructed in pSTS-MTG evoked by vowel listening, as expected from previous literature (Obleser et al., 2006; Formisano et al., 2008; Mesgarani et al., 2014), but also in IFGpTri, again in the listening task. Yet, the formant model was insufficient to explain brain activity in imagery and production. These results confirm that the superior temporal cortex represents formant structure (Formisano et al., 2008). Moreover, they suggest that *frontal* regions engage in perception, specifically encoding formant representations. However, such behavior would be modulated by auditory stimulation, despite the historical association of this region to production. Finally, our results show that phonological information, such as that provided by formants, is unique to phonological tasks, since it cannot be retrieved from tone-processing regions.

These results, while contrasting an “all-out shared” scenario for the neural code subtending vowel representation, and not fully confirming a specific “task to region” one, seem to suggest a third, more complex idea: a model based on *acoustic* properties is indeed shared between regions engaging in speech processing, but not indiscriminately (Grabski et al., 2013; Conant et al., 2018). Instead, its fundamentally acoustic nature is reflected by activity in regions engaging in a *listening* task, and with higher-level stimuli *only* (vowels, and not tones). These may contain and organize around more relevant information, like specific motor synergies (Gick and Stavness, 2013; Leo et al., 2016) of the lip-tongue complex (Conant et al., 2018): nonetheless, current limitations in the articulatory model restrict this argument, since, in our data, no production region contained articulatory information sufficient to survive statistical correction. Such discussion might, however, translate from neuroscience to phonology, by providing a finer characterization of vowel space, where apparently kinematics and acoustics do not weigh exactly the same in the brain, despite determining each other in the physics of articulation, as it is commonly taught (Stevens and House, 1955; Moore, 1992; Dang and Honda, 2002; Ladefoged and Disner, 2012).

### Formants Are Encoded in Temporal and Frontal Regions

Previous fMRI and ECoG studies already reconstructed formant space in the broad superior temporal region (Obleser et al., 2006; Formisano et al., 2008; Mesgarani et al., 2014). In line with this, we show that even a subtle arrangement of vowels in formant space holds enough information to be represented significantly in both the left pSTS-MTG and IFGpTri, during vowel listening. This presumably indicates that the temporal cortex tunes itself to the specific formant combinations of a speaker’s native language, despite its complexity. Moreover, the formant model was explained by auditory brain activity (vowel listening) in regions emerging from the listening task *only*: one may expect such behavior from regions classically involved in auditory processes, i.e., portions of the superior temporal cortex, as reported by the cited literature; instead, vowel listening also engaged the inferior frontal gyrus in our previous study (Rampinini et al., 2017), and in these results, as well, the formant model was reflected there. This suggests that a region typical to production, as the IFG is, also reflects subtle harmonic properties during vowel listening. Coming back to the hypotheses outlined in the Introduction, these results hint at a degree of code-sharing which is subtler than an all-out scenario or a specific task-to-region one: IFGpTri may perform a non-classical function, only as it “listens to” the sounds of language, retrieving acoustic information in this one specific case. The sensitivity of IFG to acoustic properties is indirectly corroborated by a study from Markiewicz and Bohland (2016), where lifting the informational weight of harmonic structure disrupted the decoding accuracy of vowels therein. The involvement of frontal regions seems consistent with other data supporting, to a certain degree, action-perception theories (Wilson et al., 2004; D’Ausilio et al., 2012a,b). On the other hand, while an interplay between temporal and frontal areas - already suggested by Luria (1966) -, is supported by computational models (Laurent et al., 2017), as well as brain data and action-perception theories, the involvement of frontal regions in listening may be modulated by extreme circumstances -as noisy or masked speech- (Adank, 2012; D’Ausilio et al., 2012b), learned stimuli over novel ones (Laurent et al., 2017), or task difficulty (Caramazza and Zurif, 1976). In this sense, IFGpTri representing auditory information may contribute to this sort of interplay. Nonetheless, our results do not provide an argument for the centrality, nor the causality of IFGpTri involvement in perception.

### Articulatory Model Fitting With Brain Activity

In phonology, the formant model is described as arising from vocal tract configurations unique to each vowel (Stevens and House, 1955; Moore, 1992; Albano Leoni and Maturi, 1995; Dang and Honda, 2002; Ladefoged and Disner, 2012). However, it has to be recognized that practical difficulties in simultaneously combining brain activity measures with linguo- and palatograms have strongly limited a finer characterization of the cerebral vowel space defined through motor markers. Indeed, to this day, the authors found scarce evidence comparing articulation kinematics with brain activity (Bouchard et al., 2016; Conant et al., 2018). Considering the articulatory model, in our data we observed how it simply never outperformed the acoustic model: in fact, it did not survive correction for multiple comparisons, even in production regions. Considering this, the formant model holds a higher signal-to-noise ratio, coming from known spectro-temporal properties, while the definition of an optimal articulatory model is still open for discussion (Atal et al., 1978; Richmond et al., 2003; Toda et al., 2008). In fact, high-dimensionality representations have frequently been derived by those reconstructing the phonatory chamber by modeling muscles, soft tissues, joints and cartilages (Beautemps et al., 2001). Such complexity is usually managed, as we did here, by means of dimensionality reduction (Beautemps et al., 2001), to achieve whole representations of the phonatory chamber. Although a vowel model described by selecting the first two formants cannot equal the richness and complexity of our articulatory model, the brain does not seem to represent the latter either, in the *pars triangularis*, or in the pSTS-MTG. Of note, a simpler, two-column articulatory model based on measures maximally correlating with F1 and F2 yielded similar results (*p* > 0.05, Bonferroni-corrected). On the other hand, we point out that our articulatory model was built upon a speaker’s vocal tract that, ultimately, was not the same as that of each single fMRI subject. Therefore, even though the formant and articulatory models *do* entertain a close relationship (signaled by elevated collinearity in our data), caution needs to be exerted in defining them as interchangeable, as shown by literature and in our results with model fitting, which favored an acoustic model in regions emerging from acoustic tasks as reported elsewhere (Cheung et al., 2016).

### Formants and Tones Do Not Overlap

The superior temporal cortex has long been implicated in processing tones, natural sounds and words using fMRI (Specht and Reul, 2003). Moreover, it seems especially probed by exquisitely acoustic dimensions such as timbre (Allen et al., 2018), harmonic structure (Formisano et al., 2008), and pitch, even when extracted from complex acoustic environments (De Angelis et al., 2018). There is also evidence of the inferior frontal cortex being broadly involved in language-related tone discrimination and learning (Asaridou et al., 2015; Kwok et al., 2016), as well as encoding timbre and spectro-temporal features in music (Allen et al., 2018), attention-based representations of different sound types (Hausfeld et al., 2018) and, in general, low-level phonological tasks, whether directly (Markiewicz and Bohland, 2016) or indirectly related to vowels (Archila-Meléndez et al., 2018). This joint pattern of acoustic information exchange by the frontal and temporal cortices may be mediated by the underlying structural connections (Kaas and Hackett, 2000) and the existence, in primates, of an auditory “what” stream (Rauschecker and Tian, 2000) specialized in resolving vocalizations (Romanski and Averbeck, 2009). Such mechanism might facilitate functional association between the frontal and temporal cortices when, seemingly, input sounds retain a semantic value for humans (recognizing musical instruments, tonal meaning oppositions, or extracting pitch from naturalistic environments for selection of relevant information). Coherently, we used tones lying within psychophysical sensitivity bands, within the frequencies of the first formant, a harmonic dimension important for vowel disambiguation, which proved to be represented across the frontal and temporal cortices (Rampinini et al., 2017). Specifically, the left STS and the bilateral IFG represented pure tones, although separate from vowels in our previous study, and here, consistently, no tone-specific region held information relevant enough to reconstruct formant, nor articulatory space. Therefore, this result hinted at the possibility of more specific organization within these hubs of sound representation.

In our previous study, the *pars triangularis* sub-perimeter coding for heard vowels also showed high accuracy in detecting tone information: in light of this, here we hypothesized the existence of a lower-to-higher-level flow of information, from sound to phoneme. Thus, when formant space was reconstructed from brain activity in the *pars triangularis* coding for heard vowels, we interpreted this result as the need for some degree of sensitivity to periodicity (frequency of pure tones) to represent harmonics (summated frequencies). Therefore, we suggest that harmony and pitch do interact, but the path is one-way from acoustics toward phonology (i.e., to construct meaningful sound representations in one’s own language), and not *vice versa*.

Interestingly, we may be looking at formant specificity as, yet again, a higher-level property retained by few selected voxels within the *pars triangularis*, spatially distinct and responsible for harmonically complex, language-relevant sounds, implying that formant space representation is featured by neurons specifically coding for phonology.

In summary, in the present study we assessed the association of brain activity with formant and articulatory spaces during listening, articulation imagery, and production of seven vowels in fMRI data. Results revealed that, as expected, temporal regions represented formants when engaged in perception; surprisingly, though, frontal regions as well encoded formants, but not vocal tract features, during vowel listening. Moreover, formant representation seems to be featured by a sub-set of voxels responsible specifically for higher level, strictly linguistic coding, since adjoining tone-sensitive regions did not retain formant-related information.

## Ethics Statement

This study was carried out in accordance with the recommendations of the relevant guidelines and regulations with written informed consent from all subjects. All subjects gave written informed consent in accordance with the Declaration of Helsinki. The protocol was approved by the Ethics Committee of the University of Pisa.

## Author Contributions

AR conceived this study, enrolled subjects, and wrote the manuscript. AR, GH, AL, and LC acquired and analyzed original data. GH and MB developed and implemented CCA analysis. AR, GH, AL, LC, and MB discussed and improved all draft versions. ER, GM, and PP supervised the study process and revised the final manuscript.

## Conflict of Interest Statement

The authors declare that the research was conducted in the absence of any commercial or financial relationships that could be construed as a potential conflict of interest.

## Footnotes

1 http://www.fon.hum.uva.nl/praat/

2 http://audacity.sourceforge.net/

3 http://www.neurobs.com/
