# Supplementary material for: Formant Space Reconstruction From Brain Activity in Frontal and Temporal Regions Coding for Heard Vowels
Source: Front Hum Neurosci. 2019 Feb 8;13:32. doi: 10.3389/fnhum.2019.00032 (PMC6383050; doi:10.3389/fnhum.2019.00032)
Supplement: Supplementary file 1 [file Data_Sheet_1.PDF]

WAV FILE DESCRIPTIONS
The following files contain the recordings of our experimental stimuli as created in Praat Vowel Editor:
1) the original speaker’s F1 and F2 values over the original speaker’s F0
2) values obtained from Canonical Correlation over the original speaker’s F0 thus to ‘reconstruct’ spoken vowels from brain activity, at group level and, for reference, in the top-performing subject(s) and region(s).

FILENAMES

behavioral_7_VWL_x_bckwd = vowels i e E a O o U with F0, F1, F2 values of the original speaker.

behavioral_U_A_I = vowels U A I with F0, F1, F2 values of the original speaker.

brain_ifg_7_VWL_x_bckwd = vowels i e E a O o U with F0 of original speaker, and F1, F2 values reconstructed from brain activity in the inferior frontal gyrus. Group level.

brain_ifg_sub_04_U_A_I = vowels U A I with F0 of original speaker, and and F1, F2 values reconstructed from brain activity in the inferior frontal gyrus of subject 04 (best performer in the region).

brain_ifg_U_A_I = vowels U A I with F0 of original speaker, and F1, F2 values reconstructed from brain activity in the inferior frontal gyrus. Group level.

brain_sts_7_VWL_x_bckwd = vowels i e E a O o U with F0 of original speaker, and F1, F2 values reconstructed from brain activity in the superior temporal sulcus. Group level.

brain_sts_sub_05_U_A_I = vowels U A I with F0 of original speaker, and F1, F2 values reconstructed from brain activity in the superior temporal sulcus of subject 05 (best performer in that region).

brain_sts_U_A_I = vowels U A I with F0 of original speaker, and F1, F2 values reconstructed from brain activity in the superior temporal sulcus. Group level.
